# Genomic data sharing for translational research and diagnostics

**DOI:** 10.1186/s13073-014-0078-2

**Published:** 2014-09-26

**Authors:** Peter N Robinson

**Affiliations:** Institute for Medical and Human Genetics, Charité-Universitätsmedizin Berlin, Augustenburger Platz 1, Berlin, 13353 Germany; Berlin Center for Regenerative Therapies (BCRT), Charité-Universitätsmedizin Berlin, Augustenburger Platz 1, Berlin, 13353 Germany; Max Planck Institute for Molecular Genetics, Ihnestrasse 63-73, Berlin, 14195 Germany; Institute for Bioinformatics, Department of Mathematics and Computer Science, Freie Universität Berlin, Takustrasse 9, Berlin, 14195 Germany

## Abstract

Translational genomics is changing, not only in the technology used but also in the sharing of data. The enormous potential for genomics technologies to improve patient care has been recognized, but it will not be reached unless powerful but secure data-sharing technologies are developed. A recent study demonstrates the power of federated queries, in which sequence variants can be searched simultaneously in files distributed over multiple centers.

## Changing practices in translational genomics

The past few years have witnessed several shifts in the practice of exome and genome sequencing. Thanks to the dramatic drop in cost and to the increasing penetration of bioinformatics into clinical medicine and translational research, numerous groups around the world have adopted these technologies and used them to identify hundreds of novel Mendelian disease genes, and next-generation sequencing (NGS) is making its way into routine clinical diagnostics. Hand in hand with these technological advances, new challenges are emerging for bioinformatics in translational genomics. Read mapping and variant calling are by no means simple, routine procedures, as recent reports on the low concordance of different variant calling pipelines [1] suggest. However, there are mature, if imperfect, algorithms available for these tasks, and widely used tools, such as the GATK best practices pipeline [2], provide high-quality variant calls. On the other hand, the field of translational bioinformatics is now confronted with numerous unsolved problems in data interpretation and integration, in knowledge generation, and in decision-support algorithms that use NGS data to help solve clinical problems.

## Data sharing for translational genomics

The enormous potential for genomics technologies to improve patient care has been recognized in fields as diverse as rare diseases, cancer, immunology, infectious diseases, prediction of medication side effects, and therapy stratification for patients with common diseases. Progress in these fields will depend on data sharing. In 2009, about a decade after the introduction of genome-wide association studies (GWASs), the term 'missing heritability' was introduced to describe the fact that only a small proportion of variance in a population attributable to additive genetic factors had been discovered by GWASs [3]. More recently, however, a series of common diseases have started to yield their secrets to GWASs with larger study sizes than those of earlier GWASs, including some with tens of thousands of patients and over 100,000 controls [4]. Theoretical considerations suggest that rare variant association studies using genome sequencing will require similar study sizes to achieve statistical power [5]. Such large numbers can rarely be achieved by a single study, so sharing of genomic data will be required to make progress in the understanding of the genetic basis of common disease, and new technologies for enabling and streamlining genomic data sharing while maintaining privacy of study participants could benefit the field greatly.

There are substantial barriers to data sharing at the scale that is needed to enable progress in genomic medicine. These include: technical issues surrounding storing terabytes, petabytes, and soon exabytes of data, and transporting at least part of the data across public and private networks; ethical and privacy issues to protect the rights of sequenced individuals; and statistical and algorithmic issues about the best ways of combining disparate datasets to address biomedical research questions. We cannot provide a comprehensive review of genomic data-sharing initiatives here, but even in the field of rare diseases, a wide variety of approaches can be discerned. For example, ClinVar intends to provide a catalog of genetic variants together with their clinical consequences, with over 120,000 variants at the time of writing [6]. DECIPHER (DatabasE of genomiC varIants and Phenotype in Humans Using Ensembl Resources) is an international community of academic departments of clinical genetics and rare disease genomics that together have deposited genomic variants and phenome data from more than 14,450 cases for which consent has been given for public display [7]. DECIPHER has facilitated over 600 publications since its inception in 2004. Since 2014, DECIPHER has integrated data sharing for sequence variants, indels and copy number variants and has a zoomable browser that enables display across the genomic scale, from nucleotide to chromosome. DECIPHER is fully searchable by phenotype, gene name, and genomic position. In 2014, an intermediate layer of data sharing was implemented, enabling DECIPHER centers to share data with nominated partners; this enables more restricted sharing, which can facilitate interpretation when consent for full data sharing has not been given. Finally, PhenomeCentral is a repository for secure data sharing targeted at clinicians and scientists working on rare disorders [8]. The website uses phenotypic clustering based on phenotypic similarity using data encoded with the Human Phenotype Ontology, which has become a widely used resource for exchanging and analyzing data in medical genetics [9]. This can be used to identify multiple individuals affected by the same disorder, allowing data from multiple centers to be integrated in the same search in an effective and secure manner.

Several ambitious new genomic initiatives are forming with the goal of enabling data sharing by fostering agreement about data standards and technical interfaces. Several such initiatives have been shown to be particularly valuable. These include the Human Genome Variation Society (HGVS), with its widely accepted guidelines for mutation nomenclature, and the Human Variome Project (HVP), which spearheads the collection, curation, interpretation, and sharing of information on variation in the human genome. Newer projects have also shown promise, including the International Rare Disease Research Consortium (IRDiRC), initially founded by the National Institutes of Health and the European Commission, which aims to promote diagnostics for all rare diseases and accelerate the development of new treatments for them; it promotes standards for data and samples and also for other areas, including ethical and regulatory procedures. IRDiRC members are funding bodies and other organizations that will promote the use of these standards by funded researchers. Another newer project is the Global Alliance for Genomics and Health (GAGH), which promotes similar goals for all areas of genomic medicine, including rare disease. One of the projects that is emerging from the GAGH is the Beacon Project, a technical interface for implementing a web service that allows limited sharing of genetic data. A genomic variant query can be conducted at a participating 'beacon' and will answer the question of whether the institution has one or more human genomes with a certain allele at a certain position (the answer is a simple 'yes' or 'no'). Thus, if a research group has identified an interesting rare variant at a certain position of the genome, it can easily find out whether other centers have additional genomes with the variant in question. At the time of writing, seven such beacons were listed at the GAGH website.

## The logistics of sharing genomic data

Current paradigms of sharing raw genomic data (such as FASTQ or BAM files) will not scale well for certain research goals, especially those that require a large amount of data to be integrated. For instance, the European Genome-phenome Archive (EGA) of the European Bioinformatics Institute (EBI) and the database of Genotypes and Phenotypes (dbGaP) of the National Center for Biotechnology Information (NCBI) are large archives for various types of genomic data. However, data access decisions are made for individual datasets, and thus it would not be easy for a user to obtain access and download 10 sets of 100 genomes each from 10 unrelated centers to perform an integrated computational analysis on all of the data.

In this issue of *Genome Medicine*, Ardeshirdavani and coworkers describe software, NGS-Logistics [10], which provides solutions to several data-sharing challenges that have not yet been sufficiently addressed by other groups. As an illustration of the potential utility of NGS-Logistics, imagine an international research consortium with 10 groups, each of which performs genome sequencing on 100 patients with a certain disease under study. Imagine that one of the ten groups identifies a mutation in a really promising candidate gene called *RPCG1*, but the statistical evidence provided by the analysis of the first group is not sufficient for publication. There are currently several options available to our imaginary consortium for making progress. It could decide to put all 1,000 genomes into a central repository, but this would require additional IT resources, and also the willingness of all 10 groups to share their data before publication. Alternatively, the first group could ask each of the other nine groups to share data on the *RPCG1* gene in each of the 100 genomes. But even if nine postdocs in the nine groups found the time to search out and copy the data, it still might be difficult for the first group to complete the analysis because of different formats or variant-calling settings being used in the other nine groups.

NGS-Logistics provides a different solution for this, in which each of the 10 centers installs the NGS-Logistics software and lets the software know where to find the 100 BAM files. This now allows each of the centers to ask a hypothesis-driven question about a gene or a variant. In the example above, the first center could use NGS-Logistics to run variant calling using its own preferred GATK parameters in the *RPCG1* gene at all 100 BAM files of all 10 centers. The variant calling is performed locally at each of the centers, and the results are collected and returned to the first center, a procedure that is known as a federated search. If the researcher at the first center thinks the results are of potential interest, NGS-Logistics can be used to request more data access. Importantly, the researcher at the first center is not allowed to download the genomes from the other centers, thus preserving privacy and respecting the intellectual property of collaborating groups. NGS-Logistics instead allows researchers to ask hypothesis-driven questions about specific variants or specific genes, such as *RPCG1* above, which can lead to targeted follow-up studies within collaborative networks. NGS-Logistics therefore may be useful to research consortia that are acquiring too much data to move from one partner to another and that for one of many reasons choose not to store their data in a central archive.

## Abbreviations

DECIPHER: Database of genomic variants and phenotype in humans using Ensembl resources
GAGH: Global alliance for genomics and health
GWAS: Genome-wide association study
NGS: Next generation sequencing
